# The ultrastructure of subgingival dental plaque, revealed by high-resolution field emission scanning electron microscopy

**DOI:** 10.1038/bdjopen.2015.3

**Published:** 2015-11-27

**Authors:** Richard Holliday, Philip M Preshaw, Leon Bowen, Nicholas S Jakubovics

**Affiliations:** 1 School of Dental Sciences, Centre for Oral Health Research, Newcastle University, Newcastle upon Tyne, UK; 2 Institute of Cellular Medicine, Newcastle University, Newcastle upon Tyne, UK; 3 Department of Physics, Durham University, Durham, UK

## Abstract

**Objectives/Aims:**

To explore the ultrastructure of subgingival dental plaque using high-resolution field emission scanning electron microscopy (FE-SEM) and to investigate whether extracellular DNA (eDNA) could be visualised in *ex vivo* samples.

**Materials and Methods:**

Ten patients were recruited who fulfilled the inclusion criteria (teeth requiring extraction with radiographic horizontal bone loss of over 50% and grade II/III mobility). In total, 12 teeth were extracted using a minimally traumatic technique. Roots were sectioned using a dental air turbine handpiece, under water cooling to produce 21 samples. Standard fixation and dehydration protocols were followed. For some samples, gold-labelled anti-DNA antibodies were applied before visualising biofilms by FE-SEM.

**Results:**

High-resolution FE-SEMs of subgingival biofilm were obtained in 90% of the samples. The sectioning technique left dental plaque biofilms undisturbed. Copious amounts of extracellular material were observed in the plaque, which may have been eDNA as they had a similar appearance to labelled eDNA from *in vitro* studies. There was also evidence of membrane vesicles and open-ended tubular structures. Efforts to label eDNA with immune-gold antibodies were unsuccessful and eDNA was not clearly labelled.

**Conclusions:**

High-resolution FE-SEM images were obtained of undisturbed subgingival *ex vivo* dental plaque biofilms. Important structural features were observed including extracellular polymeric material, vesicles and unusual open tubule structures that may be remnants of lysed cells. The application of an eDNA immune-gold-labelling technique, previously used successfully in *in vitro* samples, did not clearly identify eDNA in *ex vivo* samples. Further studies are needed to characterise the molecular composition of the observed extracellular matrix material.

## Introduction

A biofilm is a collection of microbial cells that forms on a surface or interface, and is encased within an extracellular polymeric matrix. Biofilms are abundant in humans and are responsible for many pathological processes, such as pneumonia, chronic wound infections and implant- and catheter-associated infections. Of specific clinical importance is the fact that biofilm microorganisms are distinct from their single-cell planktonic counterparts, for example, being up to 1,000 times less susceptible to antimicrobials.^1^ Wide-ranging methods for biofilm inhibition and disruption would be a huge benefit in many different clinical settings.

Dental plaque is a complex biofilm, with around 700 ‘natural colonisers’.^2^ Dental plaque is a major aetiological factor in several disease processes including dental caries and periodontal diseases. Understanding the composition and structure of dental plaque is key to developing new techniques for improving the treatment of biofilm-related pathologies in patients. Supragingival dental plaque can be visualised macroscopically *in vivo* with the naked eye, or using digital imaging techniques such as quantitative light fluorescence.^3^ Subgingival dental plaque, a major initiating aetiological factor in periodontal diseases, is much more challenging to study due to its protected location beneath the gumline.

A range of *in vitro* and *ex vivo* techniques have been utilised in studies of subgingival dental plaque biofilms. *In vitro* models vary from simple monospecies biofilms to complex high-throughput microfluidic systems.^4^ These are limited in their ability to replicate the physiological situation in the mouth as several species are unculturable, although some progress is being made in this area. For example, there have been recent reports of the axenic culture of a TM7 phylum organism^5^ and a species of the Synergistetes phylum, *Fretibacterium fastidiosum*.^6^

A series of studies from the 1960s to 1970s employed transmission electron microscopy and scanning electron microscopy (SEM) to explore the detailed structure of dental plaque both supragingivally and subgingivally. Transmission electron microscopy provided the first glimpses of extracellular matrix with material being visible between the microorganisms.^7^ SEM allowed further visualisation of the extracellular material but image resolution was limited.^8^ Conventional transmission electron microscopy and SEM techniques require a vacuum and samples must be dehydrated, which compromises the structural integrity of extracellular matrix material. Therefore, confocal laser scanning microscopy (CLSM) has generally taken over as the method of choice for biofilm research, as it provides images of biofilms in their natural hydrated state. However, CLSM has limited resolution and it is often difficult to visualise extracellular matrix structures. Natural biological materials such as extracted teeth present technical problems for CLSM due to the uneven nature of the surface and autofluorescence from the underlying substratum.

Field emission SEM (FE-SEM) differs from conventional SEM in the way it generates electrons, allowing enhanced imaging. Conventional SEM uses thermionic emission in which a filament is heated, allowing electrons to escape. FE-SEM used a field emission gun (cold cathode field emitter) in which a sharply pointed tungsten filament is placed under a huge electrical potential gradient, allowing electrons to escape. This results in many advantages including a smaller electron beam spot size that is then used to image specimens offering greater resolution and image contrast at low voltages. The additional use of electromagnetic immersion lens removes background low-energy secondary electrons from the image, thereby enhancing specimen contrast for biological samples through a highly focused beam of electrons.

Recently, FE-SEM has been used to obtain superior micrographs of *in vitro* biofilms formed by isolated oral bacteria.^9,10^ However, there is currently a lack of published FE-SEM studies of *ex vivo* specimens of complex dental plaque.

Understanding the content, structure and function of the biofilm matrix is of utmost importance in developing novel methods of biofilm control. Extracellular DNA (eDNA) has gained much attention as a potentially important component of biofilm matrices^11^ and although it has been convincingly demonstrated in model single species biofilms,^12^ by the use of an immune-gold-labelling technique, it has yet to be identified in more complex *in vivo* or *ex vivo* biofilms such as subgingival dental plaque.

The objectives of this study were (i) to explore the ultrastructure of subgingival dental plaque using FE-SEM and (ii) to investigate the potential of an *in vitro* immune-gold-labelling protocol for visualising eDNA in *ex vivo* samples.

## Materials and Methods

### Clinical sample collection

Study participants were recruited from the Periodontology consultation clinics of the Newcastle Dental Hospital. Study procedures were conducted within the Dental Clinical Research Facility at the hospital. Ethical approval was obtained from the Yorkshire and Humber Research Ethics Committee (Reference 14/YH/0145).

Potential participants were identified by the direct care team. Inclusion criteria were adult males or females 18 years or older, with capacity to give informed consent, requiring extraction of a tooth with radiographic horizontal bone loss of over 50% and mobility of grade II/III.^13^ Exclusion criteria were as follows: infectious or systemic disease that may be unduly affected by participation in the study; tooth with extensive caries or having limited structurally intact crown material; treatment with antibiotics for any medical or dental condition within 3 weeks before enrolment; participation in a dental research study within the previous 20 days.

Following informed consent, teeth were extracted using standard techniques but using the most minimally traumatic method possible. Luxator instruments were not used and extraction forceps alone were used to engage the crown. Any contact with the root surface was avoided. Extracted teeth were immediately placed into a buffer solution (1% Hank’s balanced salt solution (HBSS), Life Technologies Corporation, Paisley, UK), placed on ice, anonymized and then transferred to the laboratory for processing. In order to minimise variability, the tooth extractions were performed by, or under the supervision of, one clinician (RH). The laboratory analyses were undertaken blind to the identity of the patients (i.e., samples were identified by a unique sample number only).

### Sample preparation

The morphologies of the extracted teeth were studied and they were sectioned using a dental air turbine handpiece, under water cooling. The lateral root surface was not touched, the operator only handling the tooth by the occlusal and apical aspects. The bur was used from the furcation outwards to minimise potential disruption to the biofilm on the lateral surfaces. The divided samples were stored in cold buffer (4 °C, 1% HBSS) for a maximum of 6 h. The samples requiring immune-gold-labelling underwent the protocol described below. Following this, all samples were stabilised and fixed overnight in electron microscopy grade (EM-grade) 2% glutaraldehyde in Sorensons Phosphate Buffer (Taab Lab Equipment, Aldermaston, UK). Dehydration was performed in a graded ethanol series (30 min at 25%, 30 min at 50%, 30 min at 75%, 60 min at 100% and 60 min at 100%) followed by processing in a CO_2_-based critical point dryer (Baltec Critical Point Dryer, Leica Microsystems, Milton Keynes, UK) and mounting using silver DAG (Acheson Silver Dag, Agar scientific, Stansted, Essex, UK).

### Immuno-gold-labelling protocol

An immune-gold-labelling protocol for labelling eDNA was applied to the required samples as detailed by Barnes *et al.* ^12^ In brief, samples were blocked with 2% bovine serum albumin (Sigma-Aldrich, St Louis, MO, USA) in 1% HBSS at 4 °C for 45 min. The bovine serum albumin was removed and the primary antibody was applied (mouse anti-dsDNA monoclonal antibody (HYB331-01), Abcam, Cambridge, UK). This was placed on a shaking table for 60–90 min at 4 °C. The samples were then washed (3×) with 1% HBSS at 4 °C. The secondary antibody was then applied (donkey polyclonal secondary antibody to mouse IgG-H&L (12 nm gold), Abcam) and incubated for 90–120 min on a shaking table at 4 °C. The samples were washed three times with 1% HBSS at 4 °C before being fixed, dehydrated and processed as described above.

### Imaging

Scanning electron characterisation was obtained using a FEI Helios NanoLab Dual Beam MK 2 field emission microscope (FEI Europe, Eindhoven, The Netherlands). The biological specimens were examined in high vacuum conditions for high-resolution purposes of the study and detailed visualisation of the eDNA.

Imaging parameter’s included shortening working distances of 2–4 mm from the pole piece, together with a supplied imaging voltage between 3 and 5 kV electron volts depending on the sample. Modes of imaging were acquired using either the Everhart-Thornlet detector or through-the-lens detector to obtain secondary electron or backscatter electron images.

In order to image in high vacuum, a chromium high-resolution sputter-coating system (Cressington Scientific 328HR, Watford, UK) was used to deposit a thin electrical conductive layer for imaging. 8–15 nm of coating was used depending on the size of the specimens.

The present study was intended to be an exploratory proof of concept study. Where appropriate variables were summarised by descriptive statistics, frequencies and associations were calculated.

## Results

### Collection of periodontally affected teeth

Twelve teeth were extracted according to the study protocol. Eight were molar teeth and four were premolars. One was a first permanent molar, two were second permanent molars, five were third permanent molars, two were first permanent premolars and two were second permanent premolars. Three samples were unable to be divided due to the anatomy of the root pattern (fused roots). One sample suffered from a laboratory processing error and was not imaged. Twenty-one individual specimens were prepared and imaged using FE-SEM.

### Ultrastructural features of subgingival dental plaque

Subgingival dental plaque was not able to be visualised in two specimens, as it was obscured by soft tissue. Undisturbed subgingival dental plaque biofilm was observed in all of the other samples, demonstrating that the study protocol was effective for this purpose, and delivered subgingival dental plaque biofilm for analysis in 90% of the samples.

**Figure 1:**
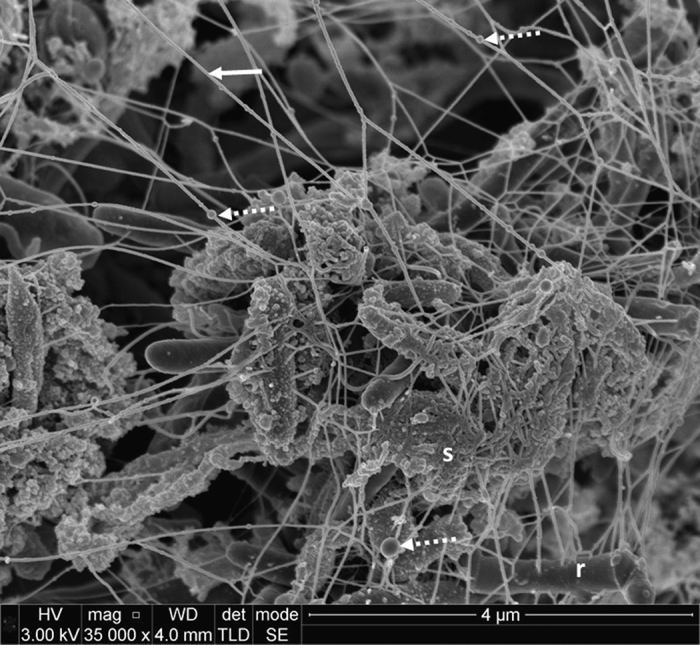
Scanning electron micrographs of subgingival dental plaque demonstrating a dense web of extracellular matrix material (solid arrow). Bead structures can be seen on the strands of extracellular material (broken arrow). Rod-shaped cells (r) were present with extracellular strands originating from the cell surface. In addition, polymeric ‘sweaters’ (s) can be seen encasing cells. ×35,000 magnification.

**Figure 2:**
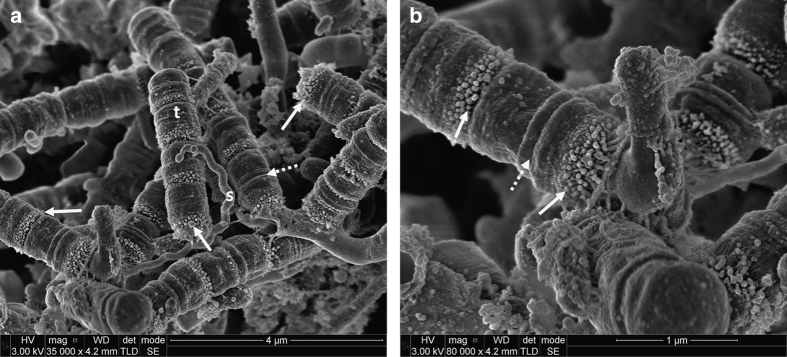
Scanning electron micrographs of subgingival dental plaque demonstrating a range of microorganisms but with the predominate species having a swollen rod morphology that could potentially be members of TM7 phylum (t). A Spirochaetal microorganism, probably *Treponema* sp., can also be seen (s). Bacterial septa can be clearly seen in the suspected TM7 microorganisms. Extensive membrane-associated vesicles can be seen associated with some septa (solid arrow), whereas other septa are totally devoid of vesicles (broken arrow). (**a**) ×35,000 magnification; (**b**) ×80,000 magnification.

**Figure 3:**
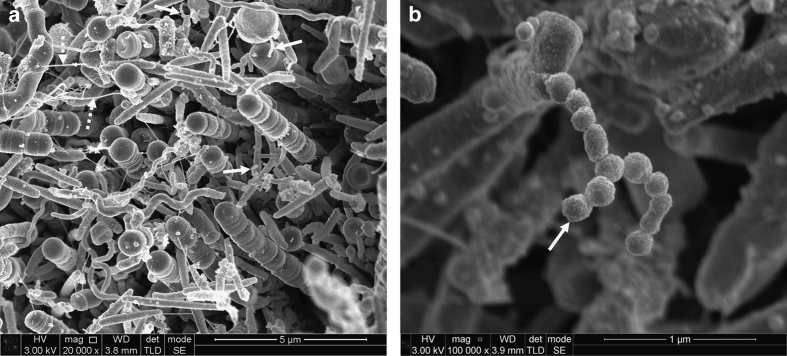
Scanning electron micrographs of subgingival dental plaque. Large coccoid cells, straight rods and spirochaetal microorganisms can be seen. Chains of vesicles (solid arrows) can be seen in several places. Fine strands of extracellular material can also be seen (broken arrow). (**a**) ×20,000 magnification; (**b**) ×100,000 magnification.

**Figure 4:**
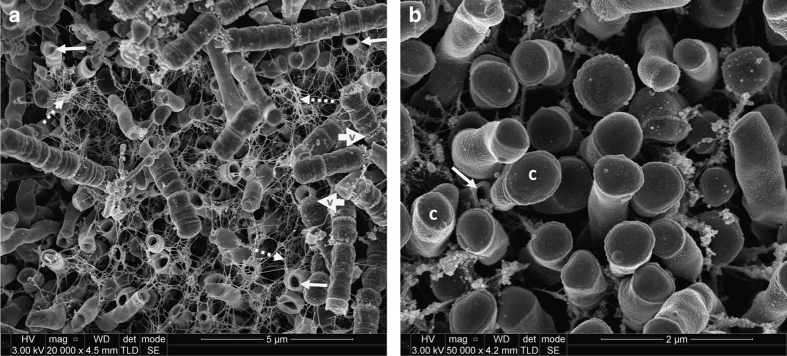
Scanning electron micrographs of subgingival dental plaque. (**a**) Numerous ‘open tubule’ structures (solid arrow). An extensive web of extracellular strands can be seen (broken arrow) encompassing the tubules and also several chains of intact short rod-shaped microorganisms. Membrane-associated vesicles can be seen (wide ‘v’ arrow). (**b**) A single ‘open tubule’ (solid arrow) surrounded by intact cells (**c**). (**a**) ×20,000 magnification; (**b**) ×50,000 magnification.

Many different bacterial cell morphologies were observed in dental plaque samples, including straight rods, spirochaetes (probably *Treponema* sp.) and chains of large short rods or coccoid cells, which may represent TM7 phylum organisms (Figures 1, 2, 3, 4). Although specific labelling methods would be required to confirm the identity of these cells, it has been previously shown that TM7 phylum organisms are abundant in subgingival plaque. For example, one study identified TM7 phylum in 96% of samples using ribosomal RNA gene profiling.^14^ These organisms may form filaments of multiple cells, ranging in length from 4 to 30 μm,^14^ which is consistent with the chains of cells observed here (Figures 1, 2, 3, 4).

In many samples abundant extracellular material was observed. In some cases this material was in the form of strands, occasionally with ‘beads’ apparent within the strands (Figure 1). The strands appeared to be originating directly from the centre of rod-shaped cells. Alternatively, the extracellular material formed a coating or mesh around bacterial cells (Figure 1).

There was evidence of vesicles associated with many of the bacterial cells in different images. These were particularly clear in the chains of large short rod-shaped cells, where vesicle structures were associated with cell division sites but not with intervening septa (Figure 2). In some cases, chains or clusters of roughly spherical particles, ~50–150 nm in diameter, were seen that are likely to be vesicles or possibly wall-less (L-form) bacteria (Figure 3). Seven samples, from five teeth, contained evidence of unusual open-ended tube structures (Figure 4). Overall, these images provide evidence of many different types of bacteria together with extensive extracellular material including strands and vesicles.

### Immune-gold labelling for eDNA

**Figure 5:**
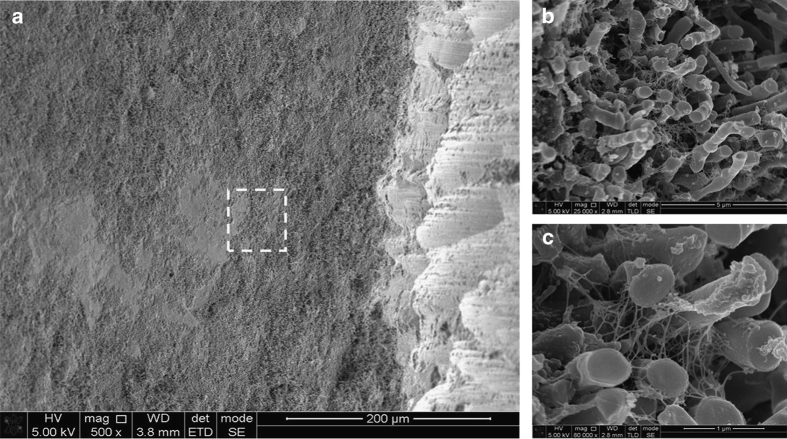
Scanning electron micrographs demonstrating intact biofilm 100 microns from a sectioned sample edge. (**a**) The cut edge of the tooth root with the gouges from the diamond bur clearly visible. The dashed box indicates where higher-resolution images were obtained. (**b**) ×25,000 magnification demonstrating intact rod-like microorganisms in a biofilm. (**c**) ×80,000 magnification showing extensive extracellular matrix as fine strands between the microorganisms.

Ten samples were prepared with the immune-gold-labelling technique. In order to control for antibody labelling, it was necessary to divide teeth into at least two sections. In the majority of tooth samples, it was possible to divide the teeth using the method outlined in the Materials and methods section to produce intact dental plaque biofilm for analysis that appeared visually to be similar to the biofilm on the undivided samples. The intact plaque was observed up to ~100 μm from a divided edge (Figure 5).

**Figure 6:**
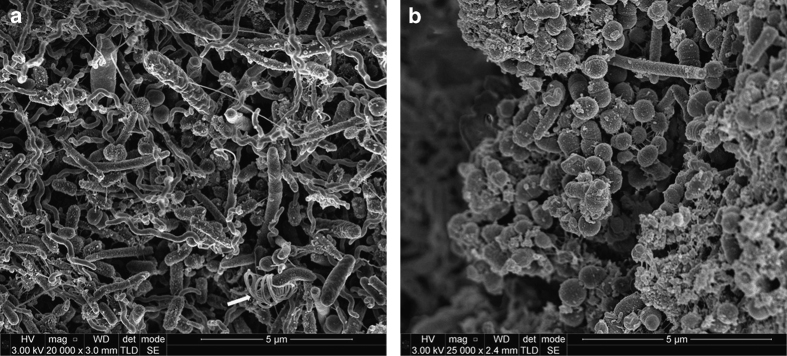
Secondary electron micrographs of subgingival dental plaque labelled with immune-gold antibodies (**a**) and control (no primary antibody) (**b**). (**a**) The complex nature of the subgingival biofilm with a range of rod- and spirochaetal-like microorganisms. A microorganism with flagella emerging from its underside can be seen (**a**, solid arrow). (**b**) Several bunches of cocci along with rod-shaped microorganisms. (**a**) ×20,000 magnification; (**b**) ×25,000 magnification.

**Figure 7:**
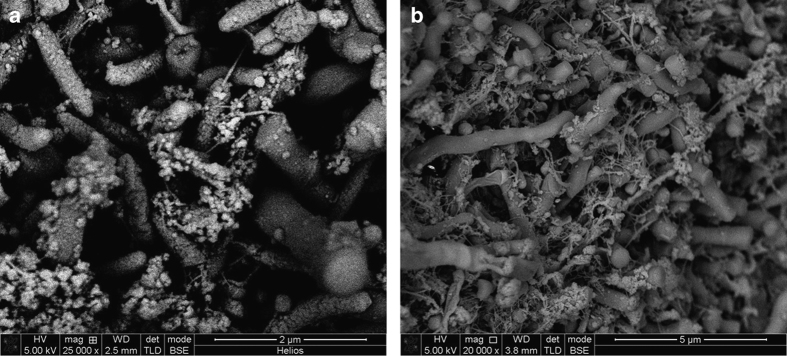
Backscatter electron micrographs of subgingival dental plaque labelled with immune-gold antibodies (**a**) and control (no primary antibody) (**b**). The labelled sample (**a**) has more contrast within the image compared with the control (**b**) but it was not possible to discern individual immune-gold particles. (**a**) ×25,000 magnification; (**b**) ×20,000 magnification.

A range of control conditions were implemented. Figure 6 demonstrates a pair of secondary electron micrographs from a single tooth that was labelled with control (no primary antibody) and with both primary and secondary antibodies. No significant differences in the distribution of gold-labelled antibodies can be seen between the samples. Figure 7 demonstrates the corresponding backscatter electron micrographs of a control and labelled sample. Again, the evidence that the antibodies were labelling eDNA specifically was not convincing. To further assess the ability of the immunogold technique to detect eDNA, controls were performed in which salmon sperm DNA was added to the SEM stub in place of the tooth sample. Even here, the backscatter images did not convincingly highlight the DNA in the samples (data not shown).

## Discussion

Biofilm matrix material has previously been observed in scanning electron micrographs of plaque from cases of advanced chronic periodontitis, in the form of fibrillar connections between organisms.^8^ However, the level of magnification and resolution of the FE-SEMs obtained in this study is much higher and indeed ultrastructural detail of the extracellular strands themselves can be observed, such as bead-like structures on the strands (Figure 1). It is not clear what these beads are, but one possibility is that they represent nucleoprotein complexes such as those that have been observed between beta toxin and eDNA in *Staphylococcus aureus* biofilms.^15^

The FE-SEM technique enables magnification and resolution that is not possible with other techniques such as CLSM. The samples are required to be dehydrated for FE-SEM and hence the biofilm will be observed in a collapsed state. Nevertheless, the micrographs demonstrate the extensive nature of the extracellular material that appears to be attached to the cell surfaces. In addition to the strands of material, some bacterial cells appeared to be covered in extracellular material. These were similar to the ‘sweater’ structures that were shown to be formed by eDNA in *E. faecalis* biofilms.^12^

Numerous examples of vesicles within the subgingival dental plaque were seen in this study, both as membrane-associated vesicles and in chain formations. Vesicles within plaque are well known and have been previously observed^7^ but not at the level of resolution seen in Figure 3. The vesicles are often loaded with DNA^16,17^ and are a proposed mechanism of eDNA release.^18,19^

The open-ended tubule structures seen in this study (Figure 4) have not been reported before to the best of our knowledge. These could be the result of natural cell lysis or of a targeted cell lysis mechanism (bacterial fratricide). Autolysis is a proposed mechanism for eDNA release and has been demonstrated in many different bacteria including *S. mutans* populations.^20^ This release allows exchange of genetic material but also potentially contributes towards the structural integrity of the biofilm. It is possible the open-ended tubules may be preparation artefacts, formed by cells rupturing during sample processing. In this case, it would appear that these cells have a structural weakness at the cell pole as the sites of rupture are remarkably consistent. It should be noted that samples were dehydrated carefully and were dried at the critical point of CO_2_, limiting the potential for cell damage. Most bacterial cells appeared to remain intact through this processing and indeed intact cells can be seen surrounding an open tubule in Figure 4b. Therefore, it seems more likely that the tubules resulted from cell lysis before fixing cells. In Figure 4a, an extensive web of extracellular stands, possibly eDNA, can be seen around the open tubules.

The general structure of the subgingival biofilm seen in our FE-SEMs is consistent with that seen with other techniques such as fluorescent *in situ* hybridisation^21^ and transmission electron microscopy^5,22^ reinforcing that any effects from sample processing were minimal.

In an attempt to confirm that eDNA was a major component of the extracellular matrix material, the immune-gold-labelling technique previously used successfully by Barnes *et al.*^12^ to demonstrate that *Enterococcus faecalis* monospecies biofilms contained eDNA was applied to 10 of our *ex vivo* samples. Despite several attempts at optimisation of the labelling protocol, we were unable to convincingly identify eDNA by immunolabelling. It is likely that the dental plaque samples contained many different types of polymer, which may have limited the access of antibodies to eDNA. Alternatively, the background material may have obscured the signal from gold-labelled eDNA. Careful controls omitting individual antibodies or using different incubation conditions failed to resolve the problems. Therefore, we feel that FE-SEM may not be appropriate for visualising gold-labelled eDNA in complex samples such as subgingival dental plaque. An alternative might be to use fluorescently labelled antibodies for CLSM analysis of eDNA in samples of hydrated plaque.

The extracellular matrix is a key structure of oral biofilms, which holds microbial cells to surfaces and protects them against external insults. It is imperative that we obtain a better understanding of the structure and composition of extracellular polymeric substances in dental plaque, in order to develop methods to degrade the matrix and improve biofilm control. The studies presented here reveal novel insights into the structure of the extracellular matrix at high resolution. It is important to note that the samples were desiccated and therefore the structures will be different from their hydrated state. Unfortunately, it was not possible to develop an immune-labelling method capable of convincingly detecting eDNA within these high-resolution FE-SEM images. We are now working on alternative methods to visualise eDNA. There is a lot of evidence that eDNA is abundant in many different biofilms^19^ and potentially eDNA could be a target for control of subgingival dental plaque, for example, by applying DNase enzymes. This could have significant implications for management of biofilm-related infections in the oral cavity, but also potentially in many other areas affected by complex biofilms throughout the human body and beyond.

## Conclusions

In conclusion, the novel imaging protocol employed in this study enabled high-resolution FE-SEM images of undisturbed subgingival *ex vivo* dental plaque biofilms to be obtained. Important structural features were observed including extracellular polymeric material, vesicles and unusual open tubule structures. The application of an eDNA immune-gold-labelling technique, previously used successfully in *in vitro* samples, did not clearly identify eDNA in *ex vivo* samples. Further development of the eDNA visualisation techniques is required in future research.
